# Executive functions as a potential neurocognitive endophenotype in anxiety disorders: A systematic review considering DSM-IV and DSM-5 diagnostic criteria classification

**DOI:** 10.1590/1980-57642015DN93000012

**Published:** 2015

**Authors:** Juliana de Lima Muller, Kamilla Irigaray Torquato, Gisele Gus Manfro, Clarissa Marceli Trentini

**Affiliations:** 1 Psychologist. Doctoral student at the Institute of Psychology, Federal University of Rio Grande do Sul, Porto Alegre RS, Brazil.; 2 Student of Psychology at the Federal University of Health Sciences of Porto Alegre, Porto Alegre RS, Brazil.; 3 PhD, Psychiatrist, Professor at the Department of Psychiatry and on the Post-graduate Program in Medical Sciences: Psychiatry, Federal University of Rio Grande do Sul, Porto Alegre RS, Brazil. Coordinator of the Anxiety Disorders Outpatient unit Program (PROTAN) of the Hospital de Clínicas de Porto Alegre and the Anxiety Disorders Program in Childhood and Adolescence (PROTAIA) of the Federal University of Rio Grande do Sul and Hospital de Clínicas de Porto Alegre, RS, Brazil.; 4 PhD, Psychologist, Professor at the Institute of Psychology, Federal University of Rio Grande do Sul, Porto Alegre RS, Brazil. Coordinator of the Núcleo de Estudos em Avaliação Psicológica e Psicopatologia (NEAPP).

**Keywords:** endophenotypes, executive function, anxiety disorders, neuropsychology

## Abstract

Evidence in the literature indicates that neurocognitive impairments may represent endophenotypes in psychiatric disorders.

**Objective:**

This study aimed to conduct a systematic review on executive functions as a potential neurocognitive endophenotype in anxiety disorder diagnosis according to the DSM-IV and DSM-5 classifications.

**Methods:**

A literature search of the LILACS, Cochrane Library, Index Psi Periódicos Técnico-Científicos, PubMed and PsycInfo databases was conducted, with no time limits. Of the 259 studies found, 14 were included in this review.

**Results:**

Only studies on obsessive-compulsive disorder (OCD) were found. The executive function components of decision-making, planning, response inhibition, behavioral reversal/alternation, reversal learning and set-shifting/cognitive flexibility were considered to be a neurocognitive endophenotypes in OCD.

**Conclusion:**

Further studies on executive functions as a neurocognitive endophenotype in other anxiety disorders are needed since these may have different neurocognitive endophenotypes and require other prevention and treatment approaches.

## Abstract (pt)

Evidências na literatura indicam que déficits neurocognitivos podem representar endofenótipos nos transtornos psiquiátricos.

**Objetivo:**

Esse estudo teve como objetivo realizar uma revisão sistemática das funções executivas como um potencial endofenótipo neurocognitivo nos transtornos de ansiedade de acordo com as classificações diagnósticas do DSM-IV e do DSM-5.

**Métodos:**

Uma pesquisa na literatura nas bases de dados LILACS, Cochrane Library, Index Psi Periódicos Técnico-Científicos, PubMed and PsycInfo foi conduzida, sem limite de tempo. Dos 259 estudos encontrados, 14 foram incluídos nessa revisão.

**Resultados:**

Somente foram encontrados estudos sobre o transtorno obsessivo-compulsivo (TOC). Os componentes das funções executivas como a tomada de decisão, planejamento, inibição de resposta, inversão comportamental/alternância, aprendizagem reversa e mudança de foco/flexibilidade cognitiva foram considerados endofenótipos neurocognitivos no TOC.

**Conclusão:**

É necessário o desenvolvimento de estudos sobre funções executivas como um endofenótipo neurocognitivo em outros transtornos de ansiedade, pois eles podem apresentar diferentes endofenótipos neurocognitivos e podem exigir abordagens de prevenção e tratamento distintas.

## INTRODUCTION

Endophenotypes have been considered an important concept in the study of neuropsychiatric diseases. It is known that there are different types of endophenotypes: neurophysiological, biochemical, endocrinologic, neuroanatomical, cognitive, and neuropsychological (including configured self-report data). Endophenotypes are intermediate measures of diseases between phenotype and genotype, and may represent simpler clues to genetic underpinnings than the disease syndrome itself, providing the decomposition or deconstruction of psychiatric diagnosis. They are associated with a candidate gene or gene region, as well as to the heritability that is inferred from relative risk for the disorder in relatives, and disease association parameters.^1^ According to this view, some criteria must be fulfilled in order to be considered an endophenotype:

[a] be associated with the disease in the population;
[b] be state-independent (manifests in an individual whether or not the illness is active);
[c] be heritable;
[d] be co-segregated with the disease;
e) be identified in unaffected first-degree relatives (UFDR) of patients at a higher rate than in the general population.^1,2^

From this perspective, there is an ongoing search in psychiatry for candidate endophenotypes that may represent vulnerability markers for disease development and lie closer to the genetic origins of the disorder.^1^ Research on this topic has focused attention on diseases such as autism,^3^ schizophrenia,^4,5^ bipolar disorder,^6^ major depressive disorder^7^ and attention deficit hyperactivity disorder.^8^ However, to date, there are few studies exploring neuropsychological endophenotypes in anxiety disorders. The Diagnostic and Statistical Manual of Mental Disorders, Fifth Edition (DSM-5), considers the following as anxiety disorders: separation anxiety disorder, selective mutism, specific phobia, social anxiety disorder (social phobia), panic disorder, agoraphobia and generalized anxiety disorder.^9^ Although the obsessive-compulsive disorder originally belonged to this group in the Diagnostic and Statistical Manual of Mental Disorders, Fourth Edition,^10,11^ it is currently classified into the obsessive-compulsive and related disorders group representing a specific new group of disorders.^9^ Moreover, posttraumatic stress disorder and acute stress disorder, both originally part of the anxiety disorders group under the DSM-IV, now belong to trauma- and stressor-related disorders, another a new group. Although obsessive-compulsive disorder, posttraumatic stress disorder and acute stress disorder are not considered anxiety disorders under the DSM-5, there is a close relationship between them and anxiety disorders.^9^

Anxiety disorders are among the most prevalent psychiatric disorders^12^ and the most frequent in Brazil.^13^ Besides presenting a high prevalence, anxiety disorders are associated to impairment in social, academic and health aspects, as well as increased suicide rates.^14^ One of the deleterious effects that may be observed in these patients is deficit in cognitive abilities, as well as in executive functions.^15,16^ The executive functions are a complex and comprehensive construct.^17^ They allow a person to guide their own behavior according to specific objectives, evaluate their efficiency and adequacy, discard ineffective strategies and maintain the most adapted ones, aiming at problem-solving in everyday functioning.^18^ This construct encompasses specific cognitive processes, for example, controlled attention, fluency, abstract thinking, self-regulation, planning, inhibitory control and cognitive shifting.^19^

Neurocognitive dysfunctions are potential endophenotype markers in different psychiatric disorders^1,20^ and are regarded to be among the most promising candidate endophenotypes.^21^ The fact that neurocognitive functions can be reliable and stable over time makes them valuable endophenotypes.^22^

With regard to research involving the evaluation of executive functions in anxiety disorders according to the DSM-IV, there are many studies evaluating patients with OCD. It has been suggested that individuals with OCD experience difficulties in planning ability,^23-25^ cognitive and motor inhibition,^20,25,26^ shifting attention,^27,28^ decision making^23,29^ and verbal fluency.^25,30^ A meta-analysis indicated that patients with OCD were significantly impaired on tasks measuring executive functions. The researchers found a relatively large effect size for planning and a moderate effect size for set-shifting ability, cognitive inhibition, verbal fluency and processing speed.^31^

On the other hand, deficits have been found in working memory,^32^ sustained attention,^33^ processing speed,^34,35^ inhibition^34,36,37^ and attentional switching^34^ in posttraumatic stress disorder (PTSD). Furthermore, findings of a meta-analysis indicated that PTSD is associated with neurocognitive deficits of a medium magnitude in attention/working memory, and processing speed, but with smaller deficits in other components of executive functions.^38^

Studies involving the evaluation of executive functions in anxiety disorders other than OCD and PTSD suggest impairments to executive functions, as well as to set-shifting abilities,^39^ verbal fluency^15^ and working memory^40,41^, in social anxiety disorder (SAD). Conversely, a systematic review indicated sparse evidence that patients with SAD have executive dysfunction, where only one out of five neuropsychological studies found significant differences between clinical and control groups.^42^

Research investigating panic disorder (PD) has found some deficits in affected individuals on divided attention,^15^ psychomotor speed,^15,16^ initiation, inhibition,^16^ working memory,^16,43^ verbal fluency and category formation.^43^ Nevertheless, research on cognitive functions in patients with PD is limited and some studies found no impairment in executive functions.^44^ Research indicates that individuals with generalized anxiety disorder (GAD) have inhibition and cognitive flexibility difficulties.^45-47^ By contrast, other researchers have failed to find deficits in GAD or in specific phobia.^15^

Some studies have described deficits in working memory,^48^ attentional components and processing speed^49^ in selective mutism.^49^ Nevertheless, there is a lack of studies on executive functions in selective mutism, as well as separation anxiety disorder.

Therefore, it can be concluded that results are inconsistent, with little clarification as to which components of executive functions may be impaired in anxiety disorders. Research results on executive functions as an endophenotype may help elucidate this issue, clarifying whether deficits in executive functions are secondary to the presence of the disorder or whether they can serve as vulnerability markers for disease development and lie closer to the genetic origins of the disorder. Furthermore, identifying those components of executive functions that can be considered vulnerability markers for the development of an anxiety disorder may assist toward prevention in at risk populations and also emphasize the importance of a better understanding of potential neurocognitive endophenotypes in anxiety disorders.

Thus, the objective of this study was to conduct a systematic review on executive functions as a potential neurocognitive endophenotype in anxiety disorders classified according to the DSM-IV and DSM-5 diagnostic criteria. Until recently, studies involving anxiety disorder samples have assessed anxiety disorders as defined by the DSM-IV. Therefore, both DSM-IV and DSM-5 anxiety disorders were included in this systematic review.^9,10^

## METHODS

**Figure 1:**
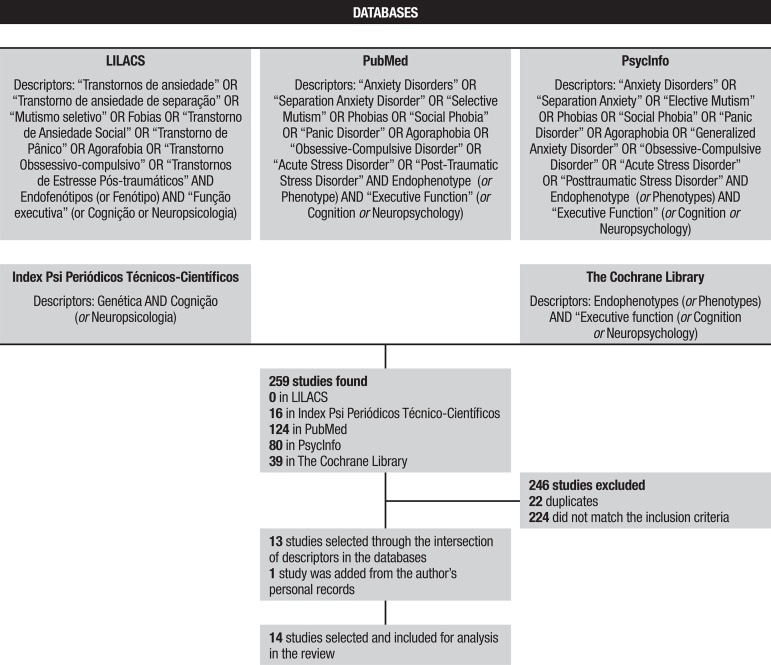
Flowchart with studies selection steps forming this systematic review.

The research question that directed this study was as follows: are executive functions a neurocognitive endophenotype in anxiety disorders, classified according to the DSM-IV or DSM-5 diagnostic criteria? In order to answer this question based on a systematic review, the Assessment of Multiple Systematic Reviews (AMSTAR) protocol was followed. The following search engines were consulted to conduct this review: LILACS, The Cochrane Library, Index Psi Periódicos Técnico-Científicos, PubMed and PsycInfo, at or around January 2015 (all research published up to this date). The descriptors were taken from DeCS (Portuguese), DeCS (English), *Terminologia em Psicologia*, MeSH and the Thesaurus of Psychological Index Terms, respectively. A search of the best descriptors to be used in each database was performed, as these differed across the databases. The descriptors used on each database can be seen in Figure 1 under the results section. For each database, the criteria "any field" was adopted, not using selection by title, author, etc.

The search and study selection were systematically and independently conducted by two investigators. Inclusion criteria were as follows:

[a] empirical studies of clinical or subclinical samples with Separation Anxiety Disorder, Selective Mutism, Specific Phobia, Social Anxiety Disorder, Panic Disorder, Agoraphobia, Generalized Anxiety Disorder, Obsessive-Compulsive Disorder, Posttraumatic Stress Disorder and/or Acute Stress Disorder, and/or with unaffected relatives;
[b] studies answering the research question, considering investigations on executive functions as a neurocognitive endophenotype in anxiety disorders; studies using at least one neuropsychological instrument to assess executive functions and/or its components;
[c] studies published in Portuguese, English or Spanish. Studies were separately examined by the investigators and excluded if they did not meet the inclusion criteria or if they were repeated. Any discordance between the investigators was discussed to reach a consensus conclusion.

## RESULTS

Based on the intersection of descriptors in the databases, the search retrieved 259 papers. The final search resulted in 13 studies analyzing components of executive functions as a possible neurocognitive endophenotype of anxiety disorders. Besides the studies selected from the databases consulted, one further paper was added from the author's personal records.^20^ Therefore, 14 studies were included in total. The flowchart is shown in Figure 1.

The 14 selected studies evaluated participants with OCD and/or their UFDR, or subclinical obsessive–compulsive participants. Table 1 shows information on the studies included in the review by country, sample, age, instruments and results. Only instruments assessing components of executive functions were included in Table 1. Also, only results that indicated deficits/impairments in components of executive functions as a neurocognitive endophenotype are shown. Instruments and results regarding other neurocognitive functions were not given in Table 1, as this was beyond the scope of the paper.

**Table 1:** Studies included in the review by country, sample, age (years), instruments and results.

Study	Country	Sample	Age (M)	Instruments	Results (deficits/impairments)*
Abramovitch et al., 2015^57^	United States	27 SOC 27 LOC	24.2 24.1	Expanded Go/No-Go Task	Response inhibition and sustained attention (SOC)
Cavedini et al., 2010^23^	Italy	35 OCD 35 UFDR 31 HC 31 HCR	35.6 45 34.7 43.2	Iowa Gambling Task, Tower of Hanoi, Wisconsin Card Sorting Test	Decision making and planning (OCD and UFDR)
Chamberlain et al., 2007^52^	United Kingdom	20 OCD 20 UFDR 20 HC	32.1 34.2 33.1	Intradimensional/Extradimensional Shift Task, Stop Signal Task, Cambridge Gamble Task	Set-shifting/cognitive flexibility and response inhibition (OCD and UFDR)
Chamberlain et al., 2008^60^	United Kingdom	14 OCD 12 UFDR 15 HC	31.7 39.5 34.8	A functional magnetic resonance imaging task capable of fractionating different components of cognitive flexibility	Reversal learning (OCD and UFDR)
Delorme et al., 2007^59^	France	64 UFDR** 47 HC	42.3 38	Tower of London Test, Trail Making Test, Design Fluency Task, Verbal Fluency Test (letter), Association Fluency task	Planning (UFDR**)
da Rocha et al., 2008^54^	Brazil	32 OCD S/Lg 17 OCD La/La	29.4 31.2	Iowa Gambling Task, CPT-II, Trail Making Test	Decision making (OCD S/Lg)
de Vries et al., 2014^61^	Netherlands	43 OCD 17 UFDR 37 HC	38.1 36.4 39.2	N-back Task	Spatial working memory (OCD and UFDR)
Lennertz et al., 2012^58^	Germany	30 OCD 30 UFDR 30 HC	40.6 42.1 42.7	Verbal Fluency Test (letter), Tower of London, Trail Making Test, Saccadic tasks	Response inhibition (OCD and UFDR)
Menzies et al., 2007^20^	United Kingdom	31 OCD 31 UFDR 31 HC	32.5 36.7 33.4	Stop Signal Task	Response inhibition (OCD and UFDR)
Rajender et al., 2011^53^	India	30 OCD 30 UFDR 30 HC	25.6 26.4 26.9	Colour Trails Test, Digit Vigilance Test, The Triads Test, Tower of London Test, Wisconsin Card Sorting Test, Stroop Test- NIMHANS version	Set-shifting/cognitive flexibility and response inhibition (OCD and UFDR)
Rao et al., 2008^50^	India	30 ROCD 30 HC	27.8 27.9	Digit span test, Continuous Performance Test, Trail Making Test, Stroop Color Word Interference Test, Wisconsin Card Sorting Test, Delayed Alternation Test, Tower of London, Controlled Oral Word Association Test, Letter Number Sequencing and Spatial Span	Set-shifting/cognitive flexibility, behavioral reversal/alternation and response inhibition (ROCD)
Viswanath et al., 2009^51^	India	25 UFDR** 25 HC	27.5 27.4	Continuous Performance Test, Trail Making Test, Stroop Colour Word Interference Test, Delayed Alternation Test, Tower of London, Controlled Oral Word Association Test, Iowa Gambling Task, Wisconsin Card Sorting Test, Letter number sequencing, Spatial span	Decision making and behavioral reversal/ alternation (UFDR**)
Zhang et al., 2015^55^	China	55 OCD 55 UFDR 55 HC	26.5 28.4 27.9	Stroop Color Word Test, Trail Making Test, Digit Span Test, Verbal Fluency Tests (letter and category), Wisconsin Card Sorting Test, Tower of London, Iowa Gambling Task, Game of Dice Task	Decision making and planning (OCD and UFDR)
Zhu et al., 2014^56^	China	14 SOC 14 LOC	19.9 19.6	Iowa Gambling Task	Decision-making (SOC)

OC: obsessive-compulsive; OCD: obsessive-compulsive disorder patients; UFDR: unaffected first-degree relatives of patients; SOC: subclinical obsessive- compulsive participants; LOC: low obsessive-compulsive symptoms control participants; HC: healthy controls; HCR: healthy controls relatives; ROCD: recovered obsessive-compulsive disorder patients; S/Lg: patients with S- and/or Lg-carriers; La/La: patients with the La/La genotype.

* Participants with deficits/impairments given in parentheses).

** Relatives of patients with obsessive-compulsive disorder.

The deficits/impairments most frequently found in OCD, were related to the following abilities: behavioral reversal/alternation,^50,51^ set-shifting/cognitive flexibility,^50,52,53^ decision making,^23,51,54-56^ response inhibition,^20,50,52,53,57,58^ planning,^23,55,59^ reversal learning,^60^ spatial working memory^59,61^ and sustained attention.^57^ For a deeper analysis of the results found in the systematic review, the neuropsychological instruments were grouped in the Discussion section according to the components of executive function evaluated.

## DISCUSSION

The purpose of this systematic review was to verify whether executive functions are a potential neurocognitive endophenotype in anxiety disorders, as diagnosed according to DSM-IV andz DSM-5 classification. Only studies on OCD were found in the systematic review, although descriptors of all anxiety disorders were used. As noted regarding research on anxiety disorders, most studies have investigated neurocognitive aspects of OCD, with few studies focusing on other anxiety disorders.^62^ This same pattern was found with regard to studies investigating executive functions as an endophenotype in anxiety disorders.

Although the 14 studies found considered OCD, they employed different methodologies and samples for the investigation of executive functions as an endophenotype of the disorder. Most of the investigations (eight studies) comprised pairs of UFDR and OCD patients compared to healthy controls,^20,23,52,53,55,58,60,61^ a design which has been used since the first studies of the endophenotype concept in psychiatry.^1^ Two studies had a similar methodology, comparing UFDR of OCD patients against healthy controls.^51,59^ One study compared OCD patients in remission versus healthy controls, investigating whether neuropsychological deficits would be present in the recovered phase.^50^ Another study evaluated OCD patients, but explored the link between decision-making and the serotonin system (serotonin transporter promoter polymorphism) in the sample,^54^ another approach to better understand the endophenotype concept.^1^ Two studies used a sampling type that has been used more recently in the study of endophenotypes. The participants of these studies were a subclinical obsessive-compulsive sample and a low obsessive-compulsive symptoms control sample.^56,57^ It has been hypothesized that the current understanding of endophenotypes in psychiatric research is that these markers lie along a continuum in the population. Concerning this hypothesis, complementary investigations in the general population are needed.^63^

It has been suggested that components of executive functions can be considered neurocognitive endophenotypes in OCD, as all the 14 studies retrieved in the systematic review indicated deficits/impairments in at least one such component. On the other hand, some components of executive functions are not linked to neurocognitive endophenotype in this disorder, such as verbal fluency, processing speed, working memory and sustained attention. Verbal fluency did not represent a vulnerability marker for development of the disease in all studies in which this component was evaluated.^50,51,55,58,59^ Some research evaluated the orthographic component (e.g. Lennertz et al., 2012)^58^ while others investigated the semantic one (e.g. Zhang et al., 2015),^55^ but in all studies verbal fluency was not suggested to be a vulnerability marker for the development of the disorder.

Processing speed was evaluated using different tests, such as the Trail Making Test - reaction time part A (e. g. Lennertz et al., 2012; Rao et al., 2008),^50,58^ Trail Making Test – reaction time part B minus reaction time part A (e.g. da Rocha et al., 2008)^54^ and Continuous Performance Test – reaction time (e.g. Rao et al., 2008).^50^ However, none of the studies that investigated this component found that it could be an endophenotype of the disorder.^50,51,54,55,58^

One study, using a visuospatial n-back task during functional magnetic resonance imaging, suggested that the working memory could be a neurocognitive endophenotype in OCD.^61^ The authors found that OCD patients and their UFDR showed task-related hyperactivity in the frontoparietal network as compared to healthy participants, providing evidence that increased recruitment of the frontoparietal network constitutes an endophenotype of the disorder.^61^ Other studies investigating working memory using the Digit Span Test, the Letter Number Sequencing and the Spatial Span presented negative findings.^50,51,55^ Thus, the majority of studies indicate that working memory is not an endophenotype in OCD.

Abramovitch et al. (2015)^57^ studied sustained attention using the Expanded Go No-Go Task (response time) to compare a subclinical obsessive-compulsive sample and a low obsessive-compulsive symptoms control sample and found that the former group had deficient sustained attention. Nevertheless, the study used a non-clinical sample and no structured clinical interview, making it difficult to extrapolate the results. Besides this study, others have assessed sustained attention with the Continuous Performance Test – omission errors (e.g. Viswanath et al., 2009),^51^ the Colour Trails Test – part 1^53^ and the Digit Vigilance Test.^53^ With the exception of Abramovitch et al. (2015),^57^ all other studies indicated that sustained attention deficits are not associated with OCD.^50,51,53,54^

On the other hand, according to this systematic review, some components of executive functions are considered neurocognitive endophenotypes in OCD. These components include the following: decision-making, planning, response inhibition, behavioral reversal/alternation, reversal learning and set-shifting/cognitive flexibility.

The most used task for the assessment of decision-making was the Iowa Gambling Task and all studies that used this test suggested that decision-making might qualify as an endophenotype for OCD.^23,51,54-56^ An interesting issue is that, in the study of da Rocha et al. (2008),^54^ this neuropsychological function was also associated with the presence of the polymorphism of the serotonin transporter gene and verified that those with the short allele (s/Lg), i.e. low expression function, performed significantly worse on the test.

As outcomes and probabilities are implicit in the Iowa Gambling Task, the participant has to initially find some effective information and figure out the options' qualities by himself by means of processing feedback of previous choices. This task assesses decision-making under ambiguity, in which the possible choices are highly ambiguous and the participant must learn to avoid the disadvantageous card decks through feedback from previous trials.^55,64^

Only two studies found intact decision-making in OCD patients and their relatives compared to healthy controls.^52,55^ One study used The Cambridge Gamble Task^52^ and the other the Game of Dice Task.^55^ The Game of Dice Task consists of a task that evaluates decision-making under risk, because explicit information about the potential consequences of different choices and their probabilities are provided in some decision situations.^65^ The study of Zhang et al. (2015)^55^ went a step further to simultaneously evaluate decision-making under ambiguity (Iowa Gambling Task) and decision making under risk situations (Game of Dice Task), and showed that dissociation of decision making under ambiguity and decision making under risk is a more appropriate potential neurocognitive endophenotype for the disorder. However, more studies involving neuropsychological instruments that assess decision making under ambiguity and decision making under risk are needed to confirm this hypothesis.

Two studies that used the Tower of London Test^55,59^ and one study that used the Tower of Hanoi Test,^23^ demonstrated that deficits in planning might represent a neurocognitive endophenotype for OCD. These findings however, are not consistent, since other studies^50,51,53,58^ also using the Tower of London Test did not indicate impairments in the groups of unaffected relatives of OCD patients or in recovered OCD patients. These studies had smaller sample sizes as compared to others,^23,55,59^ suggesting that smaller sample size may not have the power to detect differences between groups.

Considering response inhibition, Chamberlain et al. (2007)^52^ and Menzies et al. (2007)^20^ found lower performance on the Stop Signal Task (reaction times) in UFDR and OCD patients. Lennertz et al. (2012)^58^ also indicated impaired response inhibition in UFDR and OCD patients, evaluated using the anti-saccade task. Moreover, Abramovitch et al. (2015)^57^ found that a subclinical obsessive-compulsive sample committed more errors on the Expanded go/no-go task (commission errors) compared to a low obsessive-compulsive symptoms control sample. These results suggested that poor response inhibition appears to be a familial marker of OCD across the mentioned tasks.

On the other hand, the findings of two studies using the Continuous Performance Test - commission errors^50,54^ and of two studies employing the Stroop Colour Word Interference Test^51,55^ were contradictory in as far as the results did not indicate that response inhibition could be a potential neurocognitive endophenotype for the disorder. Rao et al. (2008)^50^ showed that patients in the recovered phase of the illness had significant deficits in response inhibition on the Stroop Colour Word Interference Test, but the instrument had not been validated for use in their population and language, compromising the findings observed.

Thus, it can be hypothesized that the Stop Signal Task (reaction times), the Expanded go/no-go task (commission errors) and the anti-saccade task used by Lennertz et al. (2012)^58^ appear to be more sensitive than the Continuous Performance Test (commission errors) and the Stroop Colour Word Interference Test for evaluating response inhibition as an endophenotype in OCD. Although the present systematic review showed that response inhibition represents a vulnerability marker for OCD development, impairments in this component had a relatively small effect size among patients with OCD in a recent meta-analysis.^31^ Further exploration to compare different response inhibition tests among OCD samples are needed, enabling a better understanding of the role of this component as a candidate endophenotype marker.

Behavioral reversal/alternation and reversal learning abilities were evaluated by few studies. Only two assessed behavioral reversal/alternation and used the Delayed Alternation Test.^50,51^ In this test, a rule is learnt and then subsequently needs to be inhibited and reversed in order to maintain good performance.^66^ Viswanath et al. (2009)^51^ found that unaffected relatives of OCD probands showed significant deficits on the test as compared to healthy controls whereas Rao et al. (2008)^50^ showed that patients in the recovered phase of the disorder performed poorly when compared to healthy controls i.e., deficits in behavioral reversal/alternation could be a potential endophenotype in OCD.

Reversal learning, an ability associated to behavioral flexibility after negative feedback, was evaluated in only one of the studies found in this systematic review.^60^ The authors used a functional magnetic resonance imaging task to fractionate different components of behavioral flexibility, including reversal of responses, and identified abnormally reduced activation of several cortical regions, including the lateral orbitofrontal cortex, during reversal learning in OCD patients and their unaffected relatives. The authors concluded that reversal-learning is related to hypofunction and this appeared to be a vulnerability marker for OCD. Thus, there is evidence that behavioral reversal/alternation as well as reversal learning could be considered endophenotype candidates for OCD. However, more research is needed to corroborate these findings.

Regarding set-shifting/cognitive flexibility, different instruments were used to measure these components of executive functions. Studies using the Trail Making Test (response time part B), the Design Fluency Test and the Colour Trails Test (part 2) suggested that set-shifting/cognitive flexibility are not deficient in OCD.^50,51,53,55,58,59^ Nevertheless, other studies showed contradictory results. Chamberlain et al. (2007)^52^ assessed set-shifting/cognitive flexibility with an Intradimensional/Extradimensional Shift Task and demonstrated that OCD patients and their relatives had impaired performance on these abilities. Similarly, three studies used the Wisconsin Card Sorting Test and found that deficits in set-shifting/cognitive flexibility were observed in OCD patients and their relatives^23,53^ or among patients in the recovered phase of the disease.^50^ On the other hand, two other studies used the same instrument and indicated that OCD patients and their relatives performed as well as healthy controls.^51,55^

It should be noted, however, that different versions of the Wisconsin Card Sorting Test were used by the different studies, for example, Viswanath et al. (2009)^51^ assessed their sample with a computerized version, while Zhang et al. (2015)^55^ and Rao et al. (2008)^50^ used a non-computerized version. A meta-analysis previously revealed that the use of different forms of this test might explain a significant proportion of the heterogeneity in the estimated effects for the test and that the computerized version appears to be more sensitive than the classical method in identifying deficits in patients with OCD.^31^ Thus, according to the results of this review, there is evidence that set-shifting/cognitive flexibility could be considered endophenotype candidate markers in OCD and the Wisconsin Card Sorting Test appears to be the most sensitive test for investigating these abilities. However, according to the findings of Shin et al. (2014),^31^ further studies with the computerized version could further understanding on the role of this ability as an endophenotype of OCD.

An important issue to be noted is that there was a fair degree of heterogeneity in certain variables employed by the evaluated studies. Age at disease onset was a variable indicated in only five of the studies^20,23,50,51,55^ while disease duration was also described in only five studies.^20,50,51,53,55^ These variables have previously been considered as possible moderators affecting cognitive functioning in OCD^67^ and should be better investigated in future studies.

Furthermore, the medication status and presence of comorbidities in the samples of patients differed among studies. In three studies, patients were free of medication,^53,55,61^ however, in five studies the majority or all patients were on medication.^20,50,54,58,60^ The studies of Cavedini et al. (2010)^23^ and Chamberlain et al. (2007)^52^ evaluated patients with OCD and provided no information about the use of medications. Regarding comorbidities, three studies did not exclude psychiatric comorbidities in their sample,^54,58,61^ while in seven studies the OCD patients had no comorbidities.^20,23,50,52,53,55,60^ It is possible that discrepant findings in this systematic review are attributable to confounding variables including medication status^68^ and the presence of comorbidities.^27^

Other aspects that can be attributed to the inconsistent pattern of results for some components of executive functions are the heterogeneous nature of OCD.^69^ Moreover, sample size and different test forms and methods of testing most likely influenced performance of the samples. Future studies are needed to carefully select the form of each test and the methods of testing to better investigate whether executive functions can be considered a neurocognitive endophenotype in OCD.

The investigation of endophenotypes in psychiatry is very recent^1^ and research evaluating executive functions as a neurocognitive endophenotype in OCD started even later, with the first study published in 2007.52 Thus, research assessing executive functions in patients and relatives with anxiety disorders, such as PD, GAD and SAD, could provide a better understanding of these disorders, contributing to more appropriate diagnosis and treatment of patients.

In conclusion, there are indications that decision-making, planning, response inhibition, behavioral reversal/alternation, reversal learning and set-shifting/cognitive flexibility are inherent traits of OCD. However, additional research should be conducted before definitive conclusions are reached, since few related studies have been carried out to date. Finally, through this systematic review, studies evaluating neurocognitive functions in other anxiety disorder patients besides individuals with OCD are warranted. Anxiety disorders, including OCD, have been shown to share genetic and environmental risk factors.^70^ Nevertheless, although these disorders exhibit similar features, they can have different neurocognitive endophenotypes and may require different prevention and treatment approaches. Identifying neurocognitive vulnerability markers might prove to be an important avenue toward better understanding and treatment of anxiety disorders.

## References

[1] Gottesman II, Gould TD (2003). The endophenotype concept in psychiatry: etymology and strategic
intentions. Am J Psychiatry.

[2] Leboyer M (2003). Searching for alternative phenotypes in psychiatric
genetics. Methods Mol Med.

[3] Segovia F, Holt R, Spencer M (2014). Identifying endophenotypes of autismo: a multivariate
approach. Front Comput Neurosci.

[4] Docherty AR, Coleman MJ, Tu X, Deutsch CK, Mendell NR, Levy DL (2012). Comparison of putative intermediate phenotypes in schizophrenia
patients with and without obsessive-compulsive disorder: examining evidence
for the schizo-obsessive subtype. Schizophr Res.

[5] Leppänen JM, Niehaus DJ, Koen L, Du Toit E, Schoeman R, Emsley R (2008). Deficits in facial affect recognition in unaffected siblings of
Xhosa schizophrenia patients: evidence for a neurocognitive
endophenotype. Schizophr Res.

[6] Glahn DC, Almasy L, Barguil M (2010). Neurocognitive endophenotypes for bipolar disorder identified in
multiplex multigenerational families. Arch Gen psychiatry.

[7] Peterson BS, Wang Z, Horga G (2014). Discriminating risk and resilience endophenotypes from lifetime
illness effects in familial major depressive disorder. JAMA Psychiatry.

[8] Nikolas MA, Nigg JT (2015). Moderators of neuropsychological mechanism in attention-deficit
hyperactivity disorder. J Abnorm Child Psychol.

[9] American Psychiatric Association (2014). DSM-5. Manual Diagnóstico e Estatístico de Transtornos
Mentais.

[10] American Psychiatric Association (2003). DSM-IV-TR: Manual Diagnóstico e Estatístico de Transtornos
Mentais.

[11] American Psychiatric Association (1995). 2002. DSM-IV: Manual Diagnóstico e Estatístico de
Transtornos Mentais.

[12] Kadri N, Agoub M, El Gnaoui S, Berrada S, Moussaoui D (2007). Prevalence of anxiety disorders: a population-based
epidemiological study in metropolitan area of Casablanca,
Morocco. Ann Gen Psychiatry.

[13] Andrade LH, Wang YP, Andreoni S (2012). Mental disorders in megacities: findings from the Sao Paulo
megacity mental health survey, Brazil. PloS One.

[14] Weiller E, Bisserbe JC, Maier W, Lecrubier Y (1998). Prevalence and recognition of anxiety syndromes in five European
primary care settings. A report from the WHO study on Psychological Problems
in General Health Care. Brit J Psychiatry Suppl.

[15] Airaksinen E, Larsson M, Forsell Y (2005). Neuropsychological functions in anxiety disorders in
population-based samples: evidence of episodic memory
dysfunction. J Psychiatric Res.

[16] Bolshaw M, Greca DV, Nardi AE, Cheniaux Júnior E, Fonseca RPF, Fernandez JL (2011). Funções cognitivas no transtorno do pânico:
um estudo comparativo com controles saudáveis. PSICO.

[17] Gilbert SJ, Burgess PW (2008). Executive function. Curr Biol.

[18] Malloy-Diniz LF, Sedo M, Fuentes D, Leite WB, Fuentes D, Malloy-Diniz LF, Carmargo CHP, Cosenza R (2008). Neuropsicologia das Funções
Executivas. Neuropsicologia, Teoria e Prática.

[19] Chan RC, Shum D, Toulopoulou T, Chen EY (2008). Assessment of executive functions: review of instruments and
identification of critical issues. Arch Clin Neuropsychol.

[20] Menzies L, Achard S, Chamberlain SR (2007). Neurocognitive endophenotypes of obsessive-compulsive
disorder. Brain.

[21] Cornblatt BA, Malhotra AK (2001). Impaired attention as an endophenotype for molecular genetic
studies of schizophrenia. Am J Med Genet.

[22] Rund BR (1998). A review of longitudinal studies of cognitive functions in
schizophrenia patients. Schizophr Bull.

[23] Cavedini P, Zorzi C, Piccinni M, Cavallini MC, Bellodi L (2010). Executive dysfunctions in obsessive-compulsive patients and
unaffected relatives: searching for a new intermediate
phenotype. Biol Psychiatry.

[24] Chamberlain SR, Fineberg NA, Blackwell AD, Clark L, Robbins TW, Sahakian BJ (2007). A neuropsychological comparison of obsessive-compulsive disorder
and trichotillomania. Neuropsychologia.

[25] Tukel R, Gurvit H, Ertekin BA (2012). Neuropsychological function in obsessive-compulsive
disorder. Compr Psychiatry.

[26] Abramovitch A, Dar R, Schweiger A, Hermesh H (2011). Neuropsychological impairments and their association with
obsessive-compulsive symptom severity in obsessive-compulsive
disorder. Arch Clin Neuropsychol.

[27] Aycicegi A, Dinn WM, Harris CL, Erkmen H (2003). Neuropsychological function in obsessive-compulsive disorder:
effects of comorbid conditions on task performance. Europ Psychiatry.

[28] Fenger MM, Gade A, Adams KH, Hansen ES, Bolwig TG, Knudsen GM (2005). Cognitive deficits in obsessive-compulsive disorder on tests of
frontal lobe functions. Nordic J Psychiatry.

[29] Starcke K, Tuschen-Caffier B, Markowitsch HJ, Brand M (2010). Dissociation of decisions in ambiguous and risky situations in
obsessive-compulsive disorder. Psychiatry Res.

[30] Rampacher F, Lennertz L, Vogeley A (2010). Evidence for specific cognitive deficits in visual information
processing in patients with OCD compared to patients with unipolar
depression. Prog Neuropsychopharmacol Biol Psychiatry.

[31] Shin NY, Lee TY, Kim E, Kwon JS (2014). Cognitive functioning in obsessive-compulsive disorder: a
meta-analysis. Psychol Med.

[32] Kozaric-Kovacic D, Mestrovic AH, Rak D, Muzinic L, Marinic I (2013). Cognitive status of Croatian combat veterans and their
compensation-seeking. J Forensic Psychiatry Psychol.

[33] Vasterling JJ, Duke LM, Brailey K, Constans JI, Allain AN Jr., Sutker PB (2002). Attention, learning, and memory performances and intellectual
resources in Vietnam veterans: PTSD and no disorder
comparisons. Neuropsychology.

[34] Aupperle RL, Allard CB, Grimes EM (2012). Dorsolateral prefrontal cortex activation during emotional
anticipation and neuropsychological performance in posttraumatic stress
disorder. Arch Gen Psychiatry.

[35] Cohen BE, Neylan TC, Yaffe K, Samuelson KW, Li Y, Barnes DE (2013). Posttraumatic stress disorder and cognitive function: findings
from the mind your heart study. J Clin Psychiatry.

[36] Leskin LP, White PM (2007). Attentional networks reveal executive function deficits in
posttraumatic stress disorder. Neuropsychology.

[37] Shucard JL, McCabe DC, Szymanski H (2008). An event-related potential study of attention deficits in
posttraumatic stress disorder during auditory and visual Go/NoGo continuous
performance tasks. Biol Psychol.

[38] Scott JC, Matt GE, Wrocklage KM (2015). A quantitative meta-analysis of neurocognitive functioning in
posttraumatic stress disorder. Psychol Bull.

[39] Cohen LJ, Hollander E, DeCaria CM (1996). Specificity of neuropsychological impairment in
obsessive-compulsive disorder: a comparison with social phobic and normal
control subjects. J Neuropsychiatry Clin Neurosci.

[40] Amir N, Bomyea J (2011). Working memory capacity in generalized social
phobia. J abnorm Psychol.

[41] Topçuoglu V, Fistikci N, Ekinci O, Gimzal GA, Comert AB (2009). Assessment of executive functions in social phobia patients using
the Wisconsin Card Sorting Test. Turk Psikiyatri Derg.

[42] O'Toole MS, Pedrersen AD (2011). A systematic review of neuropsychological performance in social
anxiety disorder. Nordic J Psychiatry.

[43] Castillo EP, Coy PEC, Shejet FO, Duran ET, Cabrera DM (2010). Evaluación de funciones cognitivas: atención y
memoria en pacientes con transtorno de pánico. Salud Mental.

[44] Alves MRP, Pereira VM, Machado S, Nardi AE, Silva ACO (2013). Cognitive functions in patients with panic disorder: a literature
review. Rev Bras Psiquiatr.

[45] Hazlett-Stevens H Cognitive flexibility deficits in generalized anxiety disorder.

[46] Mathews A, MacLeod C (1985). Selective processing of threat cues in anxiety
states. Behav Res Ther.

[47] Salters-Pedneault K, Suvak M, Roemer L (2008). An experimental investigation of the effect of worry on responses
to a discrimination learning task. Behav Ther.

[48] Manassis K, Tannock R, Garland EJ, Minde K, McInnes A, Clark S (2007). The sounds of silence: language, cognition, and anxiety in
selective mutism. J Am Acad Child Adolesc Psychiatry.

[49] Gray RM, Jordan CM, Ziegler RS, Livingston RB (2002). Two sets of twins with selective mutismo: neuropsychological
findings. Child Neuropsychol.

[50] Rao NP, Reddy YC, Kumar KJ, Kandavel T, Chandrashekar CR (2008). Are neuropsychological deficits trait markers in
OCD?. Prog Neuropsychopharmacol Biol Psychiatry.

[51] Viswanath B, Reddy YCJ, Kumar K J, Kandavel T, Chandrashekar CR (2009). Cognitive endophenotypes in OCD: A study of unaffected siblings
of probands with familial OCD. Prog Neuropsychopharmacol Biol Psychiatr.

[52] Chamberlain SR, Fineberg NA, Menzies LA (2007). Impaired cognitive flexibility and motor inhibition in unaffected
first-degree relatives of patients with obsessive-compulsive
disorder. Am J Psychiatry.

[53] Rajender G, Bhatia MS, Kanwal K, Malhotra S, Singh TB, Chaudhary D (2011). Study of neurocognitive endophenotypes in drug-naive
obsessive-compulsive disorder patients, their first-degree relatives and
healthy controls. Acta Psychiatr Scand.

[54] da Rocha FF, Malloy-Diniz L, Lage NV, Romano-Silva MA, de Marco LA, Correa H (2008). Decision-making impairment is related to serotonin transporter
promoter polymorphism in a sample of patients with obsessive-compulsive
disorder. Behav Brain Res.

[55] Zhang L, Dong Y, Ji Y (2015). Dissociation of decision making under ambiguity and decision
making under risk: a neurocognitive endophenotype candidate for
obsessive-compulsive disorder. Prog Neuropsychopharmacol Biol Psychiatry.

[56] Zhu C, Yu F, Ye R (2014). External error monitoring in subclinical obsessive-compulsive
subjects: electrophysiological evidence from a Gambling Task. PloS One.

[57] Abramovitch A, Shaham N, Levin L, Bar-Hen M, Schweiger A (2015). Response inhibition in a subclinical obsessive-compulsive
sample. J Behav Ther Exp Psychiatry.

[58] Lennertz L, Rampacher F, Vogeley A (2012). Antisaccade performance in patients with obsessive-compulsive
disorder and unaffected relatives: further evidence for impaired response
inhibition as a candidate endophenotype. Eur Arch Psychiatry Clin Neurosci.

[59] Delorme R, Gousse V, Roy I (2007). Shared executive dysfunctions in unaffected relatives of patients
with autism and obsessive-compulsive disorder. Eur Psychiatry.

[60] Chamberlain SR, Menzies L, Hampshire A (2008). Orbitofrontal dysfunction in patients with obsessive-compulsive
disorder and their unaffected relatives. Science.

[61] de Vries FE, de Wit SJ, Cath DC (2014). Compensatory frontoparietal activity during working memory: an
endophenotype of obsessive-compulsive disorder. Biol Psychiatry.

[62] Ferreri F, Lapp LK, Peretti CS (2011). Current research on cognitive aspects of anxiety
disorders. Curr Opin Psychiatry.

[63] Cannon TD, Keller MC (2006). Endophenotypes in the genetic analysis of mental
disorders. Ann Rev Clin Psychol.

[64] Bechara A, Damasio AR, Damasio H, Anderson SW (1994). Insensivity to future consequences following damage to human
prefrontal cortex. Cognition.

[65] Brand M, Fujiwara E, Borsutzky S, Kalbe E, Kessler J, Markowitsch HJ (2005). Decision-making deficits of korsakoff patients in a new gambling
task with explicit rules: associations with executive
functions. Neuropsychology.

[66] Chamberlain SR, Blackwell AD, Fineberg NA, Robbins TW, Sahakian BJ (2005). The neuropsychology of obsessive compulsive disorder: the
importance of failures in cognitive and behavioural inhibition as candidate
endophenotypic markers. Neurosci Biobehav Rev.

[67] Kuelz AK, Hohagen F, Voderholzer U (2004). Neuropsychological performance in obsessive-compulsive disorder:
a critical review. Biol Psychol.

[68] Segalas C, Alonso P, Real E (2010). Memory and strategic processing in first-degree relatives of
obsessive-compulsive patients. Psychol Med.

[69] Hashimoto N, Nakaaki S, Omori IM (2011). Distinct neuropsychological profiles of three major symptom
dimensions in obsessive-compulsive disorder. Psychiatry Res.

[70] Tambs K, Czajkowsky N, Roysamb E (2009). Structure of genetic and environmental risk factors for
dimensional representations of DSM-IV anxiety disorders. Brit J Psychiatry.

